# Psychosocial Determinants of Fruit and Vegetable Consumption in a Japanese Population

**DOI:** 10.3390/ijerph13080786

**Published:** 2016-08-05

**Authors:** Da-Hong Wang, Michiko Kogashiwa, Naoko Mori, Shikibu Yamashita, Wakako Fujii, Nobuo Ueda, Hiroto Homma, Hisao Suzuki, Noriyoshi Masuoka

**Affiliations:** 1 Department of Biochemistry, Okayama University of Science, 1-1 Ridai-cho, Okayama 700-0005, Japan; 2 Department of Health and Nutrition, Junior College of Shimane University, 100-205 Horo-machi, Matsue 690-0886, Japan; m-kogashiwa@water.sannet.ne.jp; 3 Department of Human Nutrition, Faculty of Human Nutrition, Seitoku University, 550 Iwase, Matsudo 271-8555, Japan; nkawano@seitoku.ac.jp (N.M.); nbo8ueda@seitoku.ac.jp (N.U.); 4 Department of Human Life, Faculty of Food and Nutrition, Okayamagakuin University, 2-28-12-201 Kugahara, Ohta, Tokyo 146-0085, Japan; shikibunn@yahoo.co.jp; 5 Department of Nutrition, Mimasaka Junior College, 50 kitazono-cho, Tsuyama 708-8511, Japan; fujii@mimasaka.ac.jp; 6 Department of Brewing and Fermentation, Junior College of Tokyo University of Agriculture, 1-1-1 Sakuragaoka, Setagaya, Tokyo 156-8502, Japan; h3homma@nodai.ac.jp; 7 Institute for Education and Student Services, Okayama University, Okayama 700-8530, Japan; hsuzuki@cc.okayama-u.ac.jp; 8 Department of Life Science, Okayama University of Science, 1-1 Ridai-cho, Okayama 700-0005, Japan; masuokan@dls.ous.ac.jp

**Keywords:** fruit, vegetables, psychosocial factors, self-efficacy, perceived barrier, attitude, responsibility

## Abstract

There is limited evidence in Japan regarding the psychosocial determinants of fruit/vegetable intake. We performed a cross-sectional study of people aged 18 years or older in four regions of Japan; 2308 (men: 1012, women: 1296) individuals who completed the questionnaires were included. We found that 24.8% of people were aware of the current recommendations for vegetables and 13.2% for fruit and that “ability to design meals” and “availability when eating outside of the home” were the most important factors related to self-efficacy and barriers to fruit and vegetable intake, respectively. People with high self-efficacy (OR: 3.16; 95% CI: 2.17, 4.60 for fruit; OR: 4.52; 95% CI: 3.08, 6.64 for vegetables) were more likely to consume more fruit and vegetables. People with high scores on attitude (OR: 1.54; 95% CI: 1.06, 2.24) and social support (OR: 1.59; 95% CI: 1.11, 2.27) were more likely to consume more fruit. People with high perceived barriers (OR: 0.69; 95% CI: 0.48, 0.98) were less likely to consume fruit. This study suggests a need to increase the general population’s awareness of the fruit and vegetable intake recommendations; facilitating positive attitudes, self-efficacy, and social support for individuals and strengthening the ability of individuals to design meals with more vegetables and fruit might be useful intervention programs.

## 1. Introduction

The health benefits of fruit/vegetable consumption are widely accepted, especially in the prevention of cancer, stroke, cardiovascular disease, and hypertension [1,2,3,4,5,6,7,8,9,10] through potential mechanisms such as antioxidant activity, modulation of detoxification enzymes, stimulation of the immune system, decrease in platelet aggregation, and alterations in the cholesterol metabolism [11]. Healthy Japan 21 (published by the Ministry of Health, Labor and Welfare) and the Guidelines for a balanced diet (from the Ministry of Agriculture, Forestry and Fisheries) recommend increasing the consumption of vegetables to 350 g or more and fruit to 200 g or more per day to prevent lifestyle-related diseases [12,13]; however, despite these recommendations vegetabkle and fruit consumption has remained low among Japanese adults. The National Health and Nutrition Survey revealed that the average daily consumption of vegetables and fruit was 271.3 g and 119.9 g, respectively, in 2011 [14] and 280.3 g and 105.2 g, respectively, in 2013 [15]. Therefore, increasing fruit and vegetable intake is one of the public health priorities in Japan.

Individual dietary behavior can be influenced by many factors, including psychosocial factors [16,17,18]. There is increasing recognition of the importance of identifying the psychosocial influences on dietary behaviors for designing effective intervention programs [19]. However, there is limited evidence from Japan that addresses the psychosocial determinants of fruit/vegetable consumption and their relations to daily consumption; only a few small-scale studies have reported positive associations of self-efficacy, social support, and economic efficiency with vegetable intake, in which the study populations were limited to university students, the elderly, and women [20,21,22,23]. Little is known about the association of attitudes, knowledge, responsibility, and perceived barriers with fruit/vegetable intake. The present study aimed to identify the psychosocial determinants of fruit and vegetable consumption in the general Japanese population and their relationship to daily consumption. Based on the results, we further suggest some strategies that might be helpful in designing future intervention programs.

## 2. Subjects and Methods

### 2.1. Participants

The study participants were conventionally recruited through: (1) college/university student classes; (2) places of employment; (3) personal networks. We recruited a total of 3179 individuals aged 18 years or older between October 2011 and June 2013 in four regions of Japan (the Chugoku region, Kinki region, Kyushu region, and Kanto region); 2308 of them (men: 1012, women: 1296) completed the questionnaires and were included in the study (response rate: 72.6%). A self-administered questionnaire survey gathered data on demographic characteristics (age, education level, employment, household members), self-reported daily fruit/vegetable intake (instructions and examples were provided in the questionnaire), and psychosocial parameters related to fruit and vegetable intake (e.g., attitudes, nutritional knowledge, responsibility, self-efficacy, social support, and perceived barriers). The present study was carried out in accordance with the Declaration of Helsinki and the study design was approved by the Ethics Committee of Okayama University Graduate School of Medicine, Dentistry, and Pharmaceutical Sciences (EKI 529). Written informed consent was obtained from all participants.

### 2.2. Self-Reported Measures

To measure the psychosocial factors related to fruit and vegetable intake, we used a questionnaire originally developed by Havas et al. [16]. The questionnaire was translated into Japanese, and inconsistencies in the translation were noted by the researchers. It was then modified and tested in a pilot study for use in the Japanese population (data not shown).

#### 2.2.1. Attitudes, Nutritional Knowledge, Responsibility, and Social Support

Attitudes toward fruit and vegetable intake were measured with five items asking questions such as “Do you think that having 100% juice or fruit in the morning is very important to me; having a green salad or another vegetable for lunch is very important to me; and having two or more vegetables for dinner is very important to me”. Each item was scored on a five-point Likert scale ranging from “agree a lot” to “disagree a lot”.

Nutritional knowledge of fruit and vegetable intake was measured with five items asking questions such as “To get the most nutrition from your vegetables, which of the following is the best way to cook them? Boil them, dip them in batter and deep fry them, stir-fry them, bake them”; and “Which combination below includes only one vegetable? Rice and green beans, soup with onion and tomatoes, stir-fry broccoli and carrot, a pumpkin croquette and tomato juice”. The participants were also asked whether they knew how much fruit and vegetables they should eat in a day; if the answer was “Yes”, they were further asked to write down the grams of fruit and vegetables they should consume per day as recommended by Healthy Japan 21 and the Guidelines for a balanced diet [12,13].

Responsibility for food shopping and preparation was evaluated by three items asking questions such as “How in charge are you of: (1) food shopping; (2) planning meals; (3) preparing food”. Each item was scored on a three-point Likert scale ranging from “not at all in charge” to “mostly or completely in charge”.

Social support was measured with three items asking questions such as “Are there other people encouraging you to: (1) buy fruit and vegetables; (2) prepare fruit and vegetables; (3) eat more fruit and vegetables”.

#### 2.2.2. Self-Efficacy

Self-efficacy was measured with 10 items asking questions such as “How sure are you that you can eat more fruit and vegetables every day; have 100% juice or fruit in the morning on most days; plan meals with more fruit and vegetables; and eat fruit and vegetables when you are in a rush”. Three-point Likert scales (confident, somewhat confident, not confident) were used to rate the confidence they had in their ability to perform each task related to fruit and vegetable intake.

#### 2.2.3. Perceived Barriers

Perceived barriers were measured with 18 items asking questions including “Eating more fruit and vegetables or eating the recommended amount of fruit and vegetables each day is difficult because I don’t like the taste of many fruits and vegetables; they cost too much; they are not always available when I eat outside of my home; we run out of them at home; my family doesn’t like them; and I think that I’m consuming enough fruit and vegetables”. Each item was scored on a five-point Likert scale ranging from “agree a lot” to “disagree a lot”.

### 2.3. Statistical Analysis

Two-group comparisons were performed using Mann-Whitney *U* test for the continuous variables and Pearson’s χ^2^ test for the categorical variables. A trend analysis was used to examine the relation of age with fruit and vegetable intake. Variables with skewed distributions were log-transformed. We also performed factor analyses to identify the underlying factor structure of fruit and vegetable consumption. To determine the reliability of the factors, the internal consistency was calculated for each construct of the resulting factors using Cronbach’s coefficient alpha. The association of psychosocial parameters with daily consumption of fruit/vegetables was analyzed by logistical regression analysis. All statistical analyses were performed using SPSS for Windows (SPSS Inc., Chicago, IL, USA).

## 3. Results

### 3.1. Characteristics of Participants

The characteristics of the participants are shown in Table 1. More than two-thirds of the participants were college/university graduates or beyond (men 71.8%, women 79.5%) and were living together with their family or others (men 66.2%, women 77.3%). The majority of the men (62.5%) and half of the women (47.7%) reported currently working, of whom 47.5% of men and 22.0% of women worked full-time.

### 3.2. The Majority of the Participants Did Not Know the Recommended Intake of Fruits and Vegetables

Of the 2308 participants, 24.8% answered that they were aware of the current recommendations for the intake of vegetables, with a significant difference between men (13.5%) and women (33.6%) (*phi* = 0.231, *p* < 0.001) (Supplementary Table S1). Among people who reported that they were aware of the current recommendations, only 73% gave a correct answer for the daily intake of vegetables (56% men vs. 78% women, *phi* = 0.209, *p* < 0.05) (Supplementary Table S2); the proportion of participants who stated that they knew the recommended daily amount of fruit intake was even lower, only 13.2% (5% men vs. 19.6% women, *phi* = 0.213, *p* < 0.001) (Supplementary Table S3), of whom only 55.4% gave a correct answer of the recommended amount (43.1% men vs. 57.9% women, *phi* = −0.111, *p* = 0.053) (Supplementary Table S4).

A trend analysis demonstrated that fruit and vegetable intake tended to increase as age increased in both men (fruits: F = 13.091, *p* < 0.01; vegetables: F = 7.587, *p* < 0.01) and women (fruits: F = 51.743, *p* < 0.01; vegetables: F = 15.780, *p* < 0.01) (Table 2). Fruit intake showed a marked increase among participants over the age of 50, whereas the amount of vegetables consumed increased among participants over age 60. We also found that more than half of the participants (men: 60.9%, women: 51.2% for vegetables; men: 60.8%, women: 50.5% for fruits) stated that they did not know how much vegetables/fruit they were eating each day.

### 3.3. Differences in Psychosocial Determinants of Fruit/Vegetable Intake between Men and Women

We also examined the differences in psychosocial variables related to fruit and vegetable intake between men and women. The results indicated that women had relatively high scores of nutritional knowledge (*r* = −0.163, *p* < 0.01), attitude (the importance of fruit and vegetable intake) (*r* = −0.145, *p* < 0.01), self-efficacy (*r* = −0.180, *p* < 0.01), and responsibility of food purchasing and preparing/meal planning (*r* = −0.395, *p* < 0.01) in comparison with men (Table 3); men had relatively high scores of perceived barriers (*r* = −0.075, *p* < 0.01) to fruit/vegetable intake than women (Table 3).

### 3.4. Perceived Self-Efficacy and Barriers to Fruit and Vegetable Intake

A factor analysis (Maximum Likelihood Method with Promax rotation of self-efficacy and perceived barriers) revealed that a 7-factor structure (two self-efficacy factors and five barrier factors) with eigenvalues >1 explained 87.4% of the total variance and was the best-fitting model; the structure contained (1) willingness to consume fruits and vegetables anytime (eight items, Cronbach’s α = 0.81); (2) ability to design meals (two items, Cronbach’s α = 0.81); (3) economic/distribution factors (six items, Cronbach’s α = 0.84); (4) family and self-preference (four items, Cronbach’s α = 0.68); (5) availability when eating outside of the home (two items, Cronbach’s α = 0.76); (6) personal judgement and awareness (two items, Cronbach’s α = 0.68); and (7) personal habits (two items, Cronbach’s α = 0.57). All the factor loadings exceeded 0.45 (Table 4), among which “ability to design meals” and “availability when eating outside of the home” were the most important factors related to self-efficacy and barriers to fruit and vegetable intake, respectively.

### 3.5. The Association of Self-Efficacy, Perceived Barriers, Attitude, Knowledge, Responsibility, and Social Support with Fruit/Vegetable Consumption

We performed a logistical analysis of people who reported daily fruit and vegetable consumption to understand the relationship between the psychosocial determinants of fruit/vegetable consumption and daily consumption. The adjusted logistical regression analysis (Table 5) showed that people in the group with moderate (OR: 2.42, 95% CI: 1.68, 3.48 for fruits; OR: 2.65, 95% CI: 1.84, 3.82 for vegetables) and high self-efficacy (OR: 3.16; 95% CI: 2.17, 4.60 for fruits; OR: 4.52; 95% CI: 3.08, 6.64 for vegetable) were more likely to consume higher amounts of fruits (≥100 g per day) and vegetables (≥150 g per day).

People with high scores on attitude (OR: 1.54; 95% CI: 1.06, 2.24) and social support (OR: 1.59; 95% CI: 1.11, 2.27) were more likely to consume higher amount of fruits (≥100 g per day). In addition, people with medium knowledge scores of vegetable consumption (OR: 1.46; 95% CI: 1.08, 1.96) were more likely to consume higher amounts of vegetables (≥150 g per day) compared with those with low knowledge scores; however, people with high knowledge scores did not show a significant association with their daily intakes of fruit/vegetable (Table 5). In contrast, people with high perceived barriers (OR: 0.69; 95% CI: 0.48, 0.98) were less likely to consume higher amounts of fruits (≥100 g per day), and people with a medium (OR: 0.66; 95% CI: 0.46, 0.95) amount of responsibility for food purchasing and preparing/meal planning were less likely to consume higher amounts of vegetables (≥150 g per day) (Table 5).

## 4. Discussion

Healthy Japan 21 began in 2000 and recommended increasing the consumption of vegetables and fruits each day for disease prevention. However, our findings noted that less than a quarter and a seventh of our participants knew the recommended daily amounts of vegetable and fruit intake, respectively, and more than half of the participants did not know how much vegetable/fruit they were eating each day. In addition, only a proportion of those who were aware of the recommendations knew the exact amount that they should eat, particularly regarding fruit intake. No literature was found regarding the public’s awareness of the recommendations for fruit and vegetable consumption in Japan. An increase in the public awareness of the importance and of the recommendations for fruit and vegetable consumption has led to a significantly positive change in fruit and vegetable consumption in the USA [24]. Our findings suggest that raising the awareness of the recommended daily intake and of individual’s present intake of vegetables and fruit is one of the top priorities for increasing vegetable and fruit consumption; this can be accomplished through, for example, the incorporation of policies, mass media, nutritional education in community settings, and social marketing strategies [25]. We also found that people under 50 years old consumed lower amounts of fruit and vegetables in both men and women than people aged over 50 years, and most respondents consumed much less than the recommended amounts of fruit and vegetables. These results were consistent with the results of Otsuka et al. [26] and the National Health and Nutrition Survey Japan, 2013 [15].

Self-efficacy is one of the main components of the social cognitive theory. Bandura indicated that people with more confidence in their performance will expect positive outcomes [27,28]. Many studies have proved that self-efficacy has a strong association with health behavior change and maintenance [18,29,30]. In comparison to those with low perceived self-efficacy for consuming fruit and vegetables, our results demonstrate that people with moderate and high self-efficacy for eating more fruit and vegetables had a 2- to 3-fold and 2- to 4-fold increase in the likelihood of consuming higher amounts of fruit (≥100 g per day) and vegetables (≥150 g per day), respectively; this indicates the importance of improving the self-efficacy of the population for increasing fruit and vegetable intake in future intervention programs.

It is worth noting that there was a gap between attitudes towards vegetable intake and the practical application of vegetable consumption in daily life. People with higher attitude scores did not necessarily consume more vegetables, although people with higher attitude scores were likely to consume a higher amount of fruit. In addition, people who reported a medium and high responsibility for purchasing food and preparing/planning meals were less likely to consume a higher amount of vegetables (≥150 g per day). Possible explanations for their lower vegetable consumption include family and self-preference, the cost of vegetables, lack of awareness of the health benefits of vegetables, and lack of awareness of easy and appetizing recipes for vegetables. Studies in South Korea [31], Costa Rica [32], Germany [33], and Thailand [34] demonstrated that dietary planning skills can increase fruit and vegetable consumption. Some respondents who were in charge of purchasing food and preparing/planning the meals might simply not know how to plan to consume more vegetables and might be unaware of easy and appetizing recipes available for vegetables. Further studies are needed to clarify this issue.

We also found a positive association of social support with fruit intake, which is consistent with a report by McSpadden et al. [35]. A recent multicenter cross-sectional study in Japan demonstrated that students living with their grandparents consumed higher amounts of fruit and vegetables compared with those not living with their grandparents [23].

The present results showed that the main perceived barriers to fruit/vegetable consumption were cost/distribution factors, availability, family- and self-preference, personal judgement and awareness, and habits. People with higher perceived barriers had a decreased likelihood of consuming higher amounts of vegetables and fruit in particular. Specifically, men reported more perceived barriers than women. Consistently, the findings of a survey on vegetable consumption in the Kanto region of Japan demonstrated that cost, availability, inconvenience, and lack of awareness of easy recipes were major barriers to vegetable consumption [36]. In 2010, Andreyeva et al. reported a systematic review of the impact of food prices on consumption based on data from 160 US-based studies, and they found “a 10% reduction in the price of fruit and vegetables would increase purchases on average by 7.0% and 5.8%, respectively” [17]. In addition, the Special Supplemental Nutrition Program for Women, Infants, and Children (WIC) showed a successful case of increasing the consumption of fruit and vegetables (including fresh, frozen or canned fruits and vegetables) in Wisconsin, USA, by introducing a voucher scheme to decrease the price of fruits and vegetables [37]. Whether this scheme is also applicable to other countries is worth examining in multilevel studies, particularly targeting low-income households. In addition, joint efforts by dieticians, the food service industry, communities, policy makers, and the mass media are needed to improve the lack of availability, awareness and convenience related to fruit/vegetable intake.

### Strengths and Limitations of the Study

An important strength of our study is that it is one of the few examining the psychosocial determinants of fruit/vegetable consumption in varying regions of Japan. The present findings will serve as a reference for designing effective intervention programs. Several limitations must also be considered. First, our study is limited by the cross-sectional nature of the study design, which cannot determine the causal relationships between psychosocial factors and fruit/vegetable consumption. Second, some reporting bias may have been introduced because the survey was carried out via a self-reported measure on fruit/vegetable intake. Nevertheless, the average daily fruit and vegetable consumption reported by our participants was very similar to the latest results of the National Health and Nutrition Survey in Japan [15]. Third, people with high knowledge scores of fruit/vegetable intakes did not show a significant association with their daily intakes, this might be due to the small sample size in this group since the confidence intervals for the association between fruit/vegetable intake and knowledge were very wide for the “high knowledge” category (Table 5). Fourth, socioeconomic status affects the quality of an individual’s diet [38]; Lord et al. reported that people with a lower socioeconomic status consumed less fruit and vegetables than those in a higher socioeconomic status [39]. Unfortunately, data on household income were not available in this study. Finally, the participants’ recruitment process was conventional and the data were only collected from four regions of Japan, which may not be representative of the general population; further studies are needed to confirm whether the patterns of psychosocial factors regarding fruit/vegetable intake found in the present study are consistent with those of other groups in the Japanese population.

## 5. Conclusions

Our findings suggest that there is an urgent need to increase the general population’s awareness of fruit and vegetable recommendations and of their current intake, particularly among young and middle-aged people. The findings also highlight the need to not only facilitate positive attitudes, self-efficacy, and social support in individuals but also to initiate joint efforts by dieticians, the food service industry, communities, policy makers, and the mass media to improve the lack of availability, awareness and convenience of fruit/vegetable intake.

## Figures and Tables

**Table 1:** Participant characteristics.

Variable	Total (*n* = 2308)	Men (*n* = 1012)	Women (*n* = 1296)
Age			
Teens	409 (17.7)	160 (15.8)	249 (19.2)
20s	926 (40.1)	421 (41.6)	505 (39.0)
30s	271 (11.7)	118 (11.7)	153 (11.8)
40s	280 (12.1)	118 (11.7)	162 (12.5)
50s	225 (9.7)	100 (9.9)	125 (9.6)
60s	128 (5.5)	68 (6.7)	60 (4.6)
70s	62 (2.7)	23 (2.3)	39 (3.0)
Non-response	7 (0.3)	4 (0.4)	3 (0.2)
Education			
≤High school	531 (23.0)	278 (27.5)	9.5)
≥College/University	1757 (76.1)	727 (71.8)	1030 (79.5)
Non-response	20 (0.9)	7 (0.7)	13 (1.0)
Residential situation			
Live alone	627 (27.2)	338 (33.4)	289 (22.3)
Live together with family or others	1672 (72.4)	670 (66.2)	1002 (77.3)
Non-response	9 (0.4)	4 (0.4)	5 (0.4)
Currently working	1250 (54.2)	632 (62.5)	618 (47.7)
Full-time job	766 (33.2)	481 (47.5)	285 (22.0)

Values in parentheses denote percentages.

**Table 2:** Daily fruit and vegetable intake across ages.

Age	Fruit (g/day)			Vegetable (g/day)		
	Total ** (*n* = 1035)	Men** (*n* = 395)	Women ** (i = 640)	Total ** (*n* = 1025)	Men ** (*n* = 394)	Women ** (*n* = 631)
Teens	68 ± 95	53 ± 90	80 ± 98	151 ± 123	152 ± 130	151 ± 118
20s	76 ± 127	70 ± 169	80 ± 94	160 ± 140	150 ± 195	167 ± 89
30s	79 ± 82	56 ± 96	93 ± 70	192 ± 113	161 ± 113	210 ± 111
40s	81 ± 82	70 ± 98	87 ± 71	183 ± 109	170 ± 113	190 ± 106
50s	128 ± 349	158 ± 573	112 ± 88	181 ± 117	143 ± 94	202 ± 123
60s	151 ± 103	128 ± 92	171 ± 109	211 ± 122	190 ± 125	226 ± 120
70s	208 ± 125	218 ± 163	203 ± 107	228 ± 208	354 ± 336	181 ± 112

Data are expressed as the mean ± SD; ** *p* < 0.01 (data were log-transformed before the trend analysis).

**Table 3:** Mean scores of psychosocial determinants of fruit/vegetable intake by gender.

Variable	Total	Men	Women
Knowledge	3.0 ± 1.0 (2308)	2.9 ± 1.0 (1012)	3.2 ± 0.9 (1296) **
Attitude	19.2 ± 4.4 (2294)	18.5 ± 4.5 (1004)	19.8 ± 4.2 (1290) **
Self-efficacy	18.6 ± 4.7 (2272)	17.6 ± 4.7 (995)	19.3 ± 4.6 (1277) **
Social support	1.2 ± 1.2 (2290)	1.3 ± 1.2 (1002)	1.2 ± 1.3 (1288)
Responsibility	6.1 ± 2.3 (2305)	5.1 ± 1.9 (1010)	6.9 ± 2.2 (1295) **
Perceived barriers	44.3 ± 11.2 (2164)	45.4 ± 11.4 (947)	43.5 ± 11.0 (1217) **

Values are the mean ± standard deviation. Values in parentheses denote the number of respondents; ** *p* < 0.01 (men vs. women by Mann-Whitney *U* test).

**Table 4:** Factors and factor loadings derived for fruit and vegetable intake.

Self-Efficacy Factors	Factor Loading
**Willingness to consume F/V anytime (Cronbach’s α = 0.81)**	
Habit of eating 2 or more servings of vegetables for dinner	0.64
I can eat fruits and vegetables even when I’m in a rush	0.63
I can eat fruits and vegetables on days when I’m at home	0.63
I eat a green salad or another vegetable for lunch most days	0.60
I eat 5 or more servings of vegetables and 200 g or more of fruit every day	0.58
I can eat more fruit and vegetables every day	0.55
I can have 100% juice or fruit in the morning on most days	0.51
I can eat fruits and vegetables on days when I’m eating away from home	0.46
**Ability to design meals (Cronbach’s α = 0.81)**	
I can plan meals with more F/V	0.92
I can prepare fruits and vegetables so that they taste good	0.75
**Perceived Barriers**	**Factor** **Loading**
**Economic/distribution factors (Cronbach’s α = 0.84)**	
Eating 5 or more servings of vegetables a day is difficult because they cost too much	0.78
Eating 5 or more servings of vegetables a day is difficult because we run out of them at home	0.77
Eating 200 g or more of fruit a day is difficult because they cost too much	0.72
Eating 200 g or more of fruit a day is difficult because we run out of them at home	0.70
Eating 5 or more servings of vegetables a day is difficult because there are few kinds available in the winter	0.56
Eating 200 g or more of fruit a day is difficult because there are few kinds available in the winter	0.50
**Family and self-preference (Cronbach’s α = 0.68)**	
My family doesn’t like fruit	0.74
My family doesn’t like vegetables	0.65
I don’t like the taste of many fruits	0.53
I don’t like the taste of many vegetables	0.48
**Availability when eating outside of the home (Cronbach’s α = 0.76)**	
Vegetables are not always available when I eat outside of the home	1.02
Fruits are not always available when I eat outside of the home	0.55
**Personal judgement and awareness (Cronbach’s α = 0.68)**	
I think I’m eating enough fruits now	0.78
I think I’m eating enough vegetables now	0.68
**Personal habits (Cronbach’s α = 0.57)**	
Having 100% juice or fruit in the morning is difficult because I don’t have such a habit	0.74
Having 100% juice or fruit in the morning is difficult because they are not filling	0.50

Factor analysis was performed by the maximum likelihood method with promax rotation of self-efficacy and barriers.

**Table 5:** Logistical regression analysis of daily fruit intake with each psychosocial parameter score.

Variable	Daily Fruit Intake ^a^		Daily Vegetable Intake ^b^	
	OR (95% CI)	*p*	OR (95% CI)	*p*
Knowledge				
Low	1.00		1.00	
Intermediate	0.81 (0.60, 1.10)	0.177	**1.46 (1.08, 1.96)**	**0.014**
High	1.70 (0.51, 5.60)	0.386	1.79 (0.56, 5.72)	0.327
Attitude				
Low	1.00		1.00	
Intermediate	1.33 (0.95, 1.86)	0.101	1.21 (0.86, 1.70)	0.271
High	**1.54 (1.06, 2.24)**	**0.022**	1.16 (0.79, 1.70)	0.458
Self-efficacy				
Low	1.00		1.00	
Intermediate	**2.42 (1.68, 3.48)**	**<0.001**	**2.65 (1.84, 3.82)**	**<0.001**
High	**3.16 (2.17, 4.60)**	**<0.001**	**4.52 (3.08, 6.64)**	**<0.001**
Social support				
Low	1.00		1.00	
Intermediate	1.10 (0.79, 1.53)	0.561	0.88 (0.63, 1.23)	0.454
High	**1.59 (1.11, 2.27)**	**0.011**	1.29 (0.90, 1.86)	0.165
Responsibility				
Low	1.00		1.00	
Intermediate	1.03 (0.72, 1.47)	0.875	**0.66 (0.46, 0.95)**	**0.026**
High	1.05 (0.67, 1.62)	0.845	0.71 (0.46, 1.11)	0.134
Perceived barriers				
Low	1.00		1.00	
Intermediate	0.76 (0.54, 1.06)	0.105	0.79 (0.56, 1.12)	0.181
High	**0.69 (0.48, 0.98)**	**0.038**	0.84 (0.59, 1.21)	0.348

^a^ Daily fruit intake as the dependent variable was categorized as <100 g and ≥100 g per day; ^b^ Daily vegetable intake as the dependent variable was categorized as <150 g and ≥150 g per day; OR (odds ratio) was adjusted for gender, age, education level, household members, and food and nutritional education; CI: confidence interval.

## Supplementary Materials

The following are available online at www.mdpi.com/1660-4601/13/8/786/s1, Table S1: Are you aware of the current recommendations for the daily intake of vegetables? Table S2: Recognition of daily amount of vegetable recommendation among people who reported that they were aware of the recommendations. Table S3: Are you aware of the current recommendations for the daily intake of fruits? Table S4: Recognition of daily amount of fruit recommendation among people who reported that they were aware of the recommendations.
