# Supplementary material for: Psychosocial Determinants of Fruit and Vegetable Consumption in a Japanese Population
Source: Int J Environ Res Public Health. 2016 Aug 5;13(8):786. doi: 10.3390/ijerph13080786 (PMC4997472; doi:10.3390/ijerph13080786)
Supplement: Supplementary file 1 [file ijerph-13-00786-s001.pdf]

Supplementary Materials: Psychosocial Determinants of Fruit and Vegetable Consumption in a Japanese Population

Da-Hong Wang, Michiko Kogashiwa, Naoko Mori, Shikibu Yamashita, Wakako Fujii, Nobuo Ueda, Hiroto Homma, Hisao Suzuki and Noriyoshi Masuoka

1. Table S1

Table S1. Are you aware of the current recommendations for the daily intake of vegetables?

Variable	Yes	No	Non-Response
Women	436 (33.6%)	856 (66.0%)	4 (0.3%)
Men	137 (13.5%)	869 (85.9%)	6 (0.59%)
Total	573 (24.8%)	1725 (74.7%)	10 (0.4%)

2. Table S2

Table S2. Recognition of daily amount of vegetable recommendation among people who reported that they were aware of the recommendations.

Variable	Right Answer (350 g)	Wrong Answer	Non-Response
Women	340 (78.0%)	93 (21.3%)	3 (0.7%)
Men	77 (56.2%)	58 (42.3%)	2 (1.5%)
Total	417 (72.8%)	151 (26.4%)	5 (0.8%)

3. Table S3

Table S3. Are you aware of the current recommendations for the daily intake of fruits?

Variable	Yes	No	Non-Response
Women	254 (19.6%)	1036 (79.9%)	6 (0.5%)
Men	51 (5.0%)	956 (94.5%)	5 (0.5%)
Total	305 (13.2%)	1992 (86.3%)	11 (0.5%)

4. Table S4

Table S4. Recognition of daily amount of fruit recommendation among people who reported that they were aware of the recommendations.

Variable	Right Answer (200 g)	Wrong Answer
Women	147 (57.9%)	107 (42.1%)
Men	22 (43.1%)	29 (56.9%)
Total	169 (55.4%)	136 (44.6%)

© 2016 by the authors; licensee MDPI, Basel, Switzerland. This article is an open access article distributed under the terms and conditions of the Creative Commons by Attribution (CC-BY) license (http://creativecommons.org/licenses/by/4.0/).
